# Identification of a Novel Homozygous *SLC34A1* Missense Mutation and a Heterozygous *SLC34A3* Deletion in an Infant with Nephrocalcinosis, Failure to Thrive, and Hypercalcemia

**DOI:** 10.3390/ijms26178541

**Published:** 2025-09-02

**Authors:** Glorián Mura-Escorche, Leire C. García-Suarez, Isis Lebredo-Álvarez, Elena Ramos-Trujillo, Felix Claverie-Martin

**Affiliations:** 1 Research Unit, Hospital Universitario Nuestra Señora de Candelaria, 38010 Santa Cruz de Tenerife, Spain; gmuraesc@ull.edu.es; 2 Pediatrics Service, Hospital General de Fuerteventura, 35600 Puerto del Rosario, Spain; lgarsuaw@gobiernodecanarias.org; 3 Clinical Analysis Laboratory, Hospital General de Fuerteventura, 35600 Puerto del Rosario, Spain; ilebalv@gobiernodecanarias.org; 4 Nutrition and Bromatology Section, Department of Chemical Engineering and Pharmaceutical Technology, Universidad de La Laguna, 38200 San Cristóbal de La Laguna, Spain

**Keywords:** mutations, exome analysis, *SLC34A1*, *SLC34A3*, phosphate cotransporters, idiopathic infantile hypercalcemia, bioinformatics predictions

## Abstract

Renal phosphate transporters NaPi-IIa (*SLC34A1*) and NaPi-IIc (*SLC34A3*) play a crucial role in phosphate reabsorption in the proximal tubule. Biallelic loss-of-function variants in *SLC34A1* and *SLC34A3* cause two rare phosphate-wasting tubulopathies: idiopathic infantile hypercalcemia (IIH) and hereditary hypophosphatemic rickets with hypercalciuria, respectively. The phenotypes associated with these diseases are highly variable and sometimes overlap. Here, we report a rare case of a six-month-old girl of consanguineous parents with symptoms related to these diseases, including failure to thrive, nephrocalcinosis, hypercalcemia, hypophosphatemia with low TRP, elevated levels of 1,25-(OH)_2_D_3_, and suppressed PTH. An exome sequencing analysis was carried out to determine the genetic variants associated with her disease. Bioinformatics tools were used to assess variant pathogenicity. We identify a novel homozygous mutation in the *SLC34A1* gene, c.1361C>T; p.(T454M), and a previously described heterozygous *SLC34A3* 101 bp deletion. Mutation p.(T454M) affects transmembrane domain 5 of the NaPi-IIa protein, which is involved in substrate binding, probably impairing phosphate transport. Our results suggest the diagnosis of IIH type 2 in our patient and highlight the importance of exome analysis in diagnosing these tubulopathies. We suggest that the coexistent heterozygous *SLC34A3* deletion could increase the risk of renal calcifications and the severity of other symptoms.

## 1. Introduction

**Figure 1:**
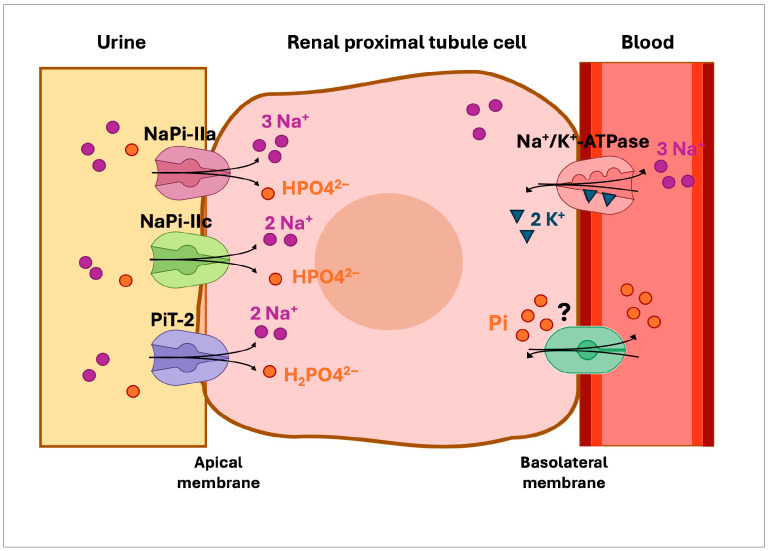
Phosphate transport in the renal proximal tubule. Sodium-dependent phosphate cotransporters, NaPi-IIa and NaPi-IIc, expressed in the apical membrane of epithelial cells reabsorb most of the filtered phosphate. A third cotransporter, PiT-2, is also found in the apical membrane but its role in the kidney is not clear. The mechanism responsible for Pi export from the basolateral membrane remains unclear, but it is hypothesized to involve Pi exchange with extracellular anions [15]. The question mark indicates that the basolateral phosphate transporter has not yet been identified.

Phosphates are involved in numerous cellular processes, including bone and teeth mineralization, regulation of protein function and intracellular signal transduction. Also, they are essential components of cell membranes, high-energy compounds and nucleic acid helical structures [1]. Therefore, the serum levels of phosphate in the body are under an intense regulation that is mediated by an interplay between intestinal absorption, storage in bones, and renal reabsorption/excretion [2]. In the kidney, free inorganic phosphate (Pi) is filtered in the glomerulus and then reabsorbed in the proximal tubule [2]. Two sodium-dependent phosphate cotransporters, NaPi-IIa and NaPi-IIc, expressed almost exclusively in the apical membrane of epithelial proximal tubule cells are accountable for the reabsorption of most (70–80%) of the filtered phosphate [3,4,5,6] (Figure 1). A third cotransporter, Pit-2, is expressed in many different epithelia including the proximal tubule, but its role in kidney is not well understood [7,8]. These transporters belong to the solute carrier (SLC) superfamily, and are encoded by the *SLC34A1, SLC34A3* and *SLC20A2* genes, respectively. Studies in mice suggest that sodium phosphate cotransporter NaPi-IIa is responsible for approximately 70% of the reabsorption of phosphate in kidneys, while NaPi-IIc accounts for less than 30% [9,10,11]. On the other hand, NaPi-IIc seems to be a growth-related renal sodium phosphate cotransporter; it is highly expressed in the weaning kidney but in adults its role is diminished [3,11]. NaPi-IIa and NaPi-IIc expression is tightly regulated by parathyroid hormone (PTH), fibroblast growth factor 23 (FGF23) and dietary phosphate intake [12,13,14,15].

Loss-of-function variants in *SLC34A1* and *SLC34A3* are the cause of two rare phosphate-wasting disorders, idiopathic infantile hypercalcemia type 2 (IIH, OMIM #616963) [16,17] and hereditary hypophosphatemic rickets with hypercalciuria (HHRH, OMIM #241530) [18,19,20], respectively. According to Orphanet (https://www.orpha.net/) (accessed on: 21 August 2025) the estimated prevalence of IIH is <1/1.000.000. The IIH type 1 (OMIM #143880), associated with mutations in the cytochrome P450 family 24 subfamily A member 1 gene (*CYP24A1*), had been described earlier [21]. This mitochondrial enzyme is implicated in the catabolism of vitamin D. Pediatric IIH type 1 and IIH type 2 patients with homozygous or compound heterozygous mutations present a common clinical phenotype characterized by hypophosphatemia, hypercalcemia, hypercalciuria, polyuria, failure to thrive, and then nephrocalcinosis or nephrolithiasis [16,17,21,22]. There are no evident clinical features that can be used to differentiate both IIH types at presentation. However, biallelic mutations in the *CYP24A1* and *SLC34A1* genes have a completely different mechanism leading to hypercalcemia. The *CYP24A1* defects cause hypercalcemia by directly blocking degradation of 1,25-(OH)_2_D_3_, which results in accumulation of active forms of vitamin D_3_ and enhancement of intestinal calcium absorption and bone reabsorption [21]. In contrast, the *SLC34A1* alterations trigger hypercalcemia because of primary renal phosphate loss followed by an improper activation of vitamin D [16]. The characteristic biochemical alterations of IIH lead to abnormal renal calcium accumulations with the resultant higher risk of nephrolithiasis and nephrocalcinosis. On the other hand, the IIH signs and the clinical differences between infants and adults depend on several environmental factors including diet, lifestyle, vitamin D intake, and sunlight exposure [23]. Disease symptoms may improve in IHH patients after infancy, independent of treatments [22]. The phenotypes associated with these three diseases are highly variable and sometimes overlap, making diagnostic particularly challenging [22,24,25]. HHRH patients present similar symptoms but often develop rickets that are not detected in IIH patients. In contrast to IIH, HHRH manifestations continue into adulthood. Patients with homozygous or compound heterozygous variants in both genes show elevated levels of 1,25-(OH)_2_D_3_, alkaline phosphatase, suppressed PTH, and hypercalciuria [22]. Heterozygous carriers show similar but less evident phenotypes. Both IIH and HHRH were originally described as autosomal-recessive disorders; however, heterozygous mutations in *SLC34A1* and *SLC34A3* seem to be associated with an increased risk to develop nephrolithiasis in adults [17,26,27].

In the present study, we describe the clinical and genetic characteristics of an infant with nephrocalcinosis, failure to thrive, and hypercalcemia. A novel homozygous missense mutation of the *SLC34A1* gene and a known *SLC34A3* deletion were identified, which could explain the urinary loss of phosphate in the patient.

## 2. Results

### 2.1. Clinical Case

**Figure 2:**
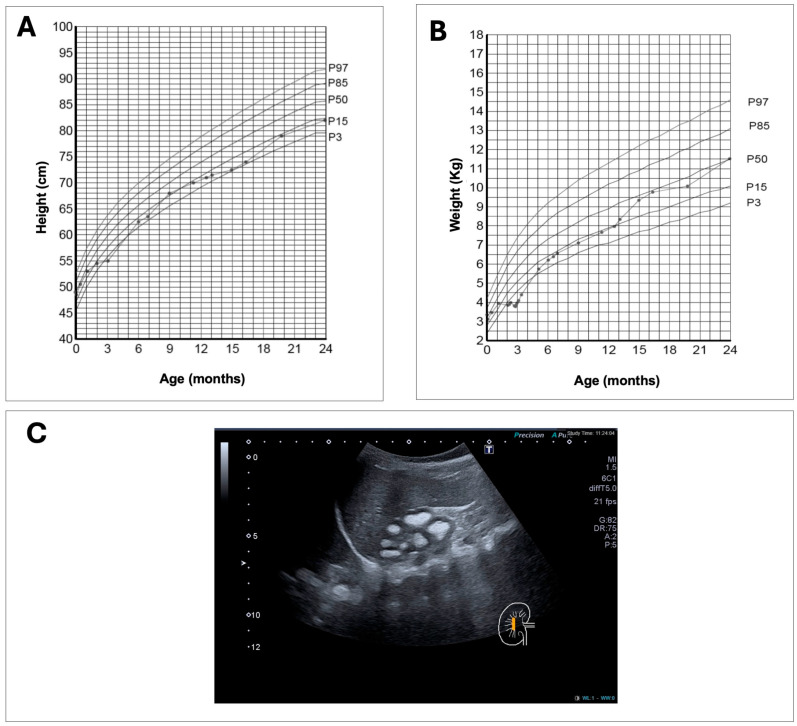
Growth parameters and renal imaging of the patient. The height (**A**) and weight (**B**) curves are shown (according to WHO standards). (**C**) Renal ultrasonography showing severe medullary nephrocalcinosis. A scale bar in centimeters is shown on the left-hand side of the figure.

The probands was a 6-month-old female infant, the first child of consanguineous parents (maternal grandmother and paternal grandfather were second cousins) from the Canary Islands. All her great-grandparents were also from the Canary Islands. She was under follow-up by her pediatrician due to failure to thrive since she was one month old. She was the result of a controlled gestation, with normal ultrasounds. The delivery was normal at 40 weeks, with an adequate weight (3350 g, p63) and a height of 49 cm (p43). There were no perinatal incidents, except for the detection, following neonatal screening, of a heterozygous mutation for sickle cell anemia. The infant maintained a weight within the 15th–50th percentile during the first month and subsequently began to lose weight until 3 months of age (<P1, −3.12 SD at the third month), with an impact on height (P2, −2.12 SD) (Figure 2A,B). In this context, leukocyturia was detected on a urine dipstick, and cefuroxime treatment was started, with very good weight regain (p9 at 6 months of age). Given the suspicion of a urinary tract infection, a renal ultrasound was ordered, which showed grade III nephrocalcinosis. Physical examination revealed marked hypotonia (head support was not achieved at 6 months), which improved a few weeks after the start of rehabilitation sessions. Persistent constipation and polyuria were also mentioned. Laboratory tests revealed creatinine within reference values, with abnormalities in phosphocalcium metabolism: hypercalcemia with plasma phosphate at the lower limit for her age and a low tubular phosphate reabsorption rate (TPR), elevated 1,25-(OH)_2_D_3_ and low PTH, variable calcium in the urine, and elevated glomerular filtration rate (GFR) and elevated urinary volume per 100 mL of glomerular filtrate (V/GF) (Table 1). The rest of the metabolic workup for nephrolithiasis/nephrocalcinosis (citraturia, oxaluria, urine amino acids, etc.) was normal. Vitamin D supplementation was discontinued at 9 months of age, and the patient was instructed not to overindulge in dairy products (no more than 400–500 mL per day). The hypercalcemia was mild throughout (at the upper limit of normal for an infant, whose primary food source is milk), with no significant hypercalciuria. Furthermore, due to the nephrocalcinosis, the patient was advised to maintain adequate hydration (2–3 L/m^2^, 1–1.5 L of water per day) and consume citrus fruits daily, while avoiding excessive salt or animal protein intake. Constipation improved after 12 months. The analysis of a genetic panel for nephrolithiasis/nephrocalcinosis was requested. Following the results, at 22 months of age, oral phosphate solution (20 mg/mL) supplementation was started, initially at 10 mg/kg/day every 6–8 h, with a progressive increase to 40 mg/kg/day. There were no significant biochemical or clinical changes after starting treatment, although parents acknowledged difficulties with adherence (due to the high number of doses). Until 24 months of age, the patient maintained a good growth rate, reaching p10-15, but below her genetic target height (167.5 cm, p75 cm), with no clinical or radiological signs of rickets. Her weight was normal (p25–50) (Figure 2A,B). Her psychomotor development was adequate, except for the generalized hypotonia, which progressively improved; she began walking at 12 months, at which point her rehabilitation follow-up ended. Subsequent ultrasound examinations revealed unchanged medullar nephrocalcinosis (Figure 2C). At two years of age, a metabolic bone series including imaging studies showed no indications of rickets or other bone defects. Although both parents were asymptomatic, they were referred to the adult Nephrology Service for evaluation. Blood tests, 24 h urine tests and renal ultrasounds were performed on both patient’s parents. The results showed that the father had decreased plasma phosphate levels (1.8 mg/dL; normal range: 2.5 to 4.5 mg/dL), a urinary phosphate level at the normal upper limit (1205 mg/24 h; normal range 100–1300 mg/24 h), and no other biochemical abnormalities. No laboratory abnormalities were found in the mother. Renal ultrasounds of either parent did not reveal any abnormalities.

### 2.2. Genetic Findings

**Figure 3:**
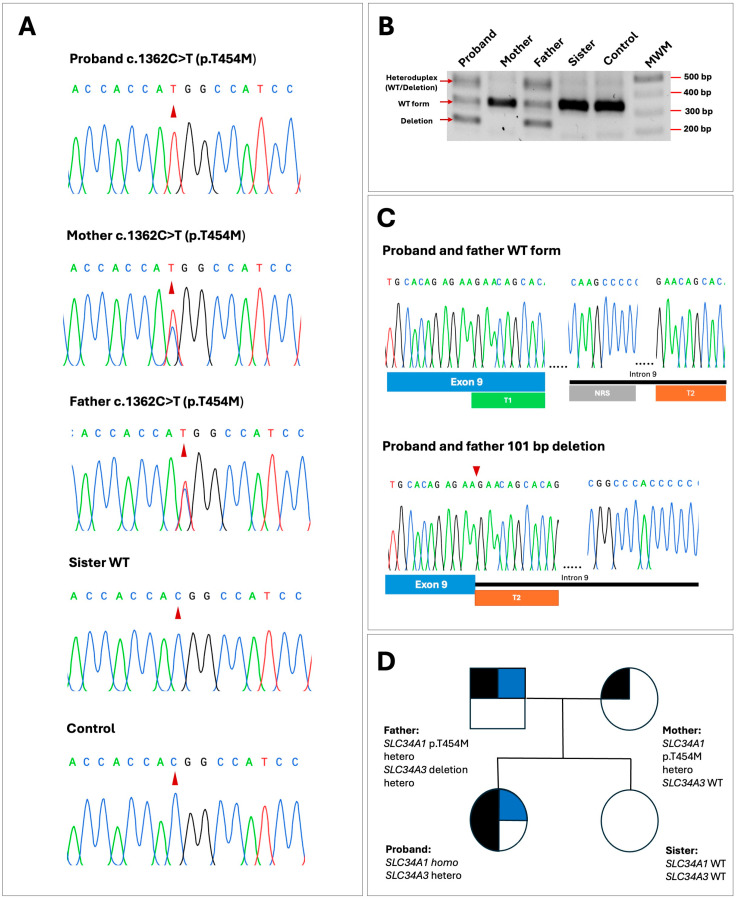
Genetic analysis of the patient and her relatives, and family pedigree. (**A**) Electropherograms of proband and family members showing the *SLC34A1* homozygous variant, c.1362C>T; p.(T454M), in the patient confirming the result of the exome analysis. Both parents present this mutation in heterozygosis. Red arrows indicate the affected nucleotide positions. (**B**) Agarose gel electrophoresis of PCR amplified products containing the *SLC34A3* region between exons 9 and 10 in family members. A band of 324 bp corresponding to the control is observed in all the family members. A smaller band of approximately 223 bp corresponding to the 101 bp deletion was also observed in the proband and her father. The higher band seen in the proband and his father is probably the result of heteroduplex formed between the normal band and the deletion. MRW, molecular weight marker. (**C**) Electropherograms and a schematic representation of the WT *SLC34A3* sequence and the 101 bp deletion identified in the proband and her father. The region between exon 9 (blue box) and intron 9 (black line) contains two direct repeats; tandem 1 (T1), green box, and tandem 2 (T2), orange box. Between these two repeats there is a non-repeat sequence (NRS, gray box). (**D**) Family pedigree illustrating the segregation of variants in the *SLC34A1* and *SLC34A3* genes. Squares represent males and circles represent females. Each symbol is divided into quadrants to indicate multiple diagnoses; black shading indicates the presence of the p.(T454M) variant in the *SLC34A1* gene, while blue shading indicates the presence of the c.925+20_926-48del deletion in *SLC34A3*. Unshaded quadrants indicate the absence of the corresponding variant.

The exome sequencing analysis of the affected member identified two potentially pathogenic variants; a novel homozygous missense variant, c.1361C>T; p.(T454M), located in exon 12 of the *SLC34A1* gene, and a heterozygous 101 bp deletion in intron 9 of *SLC34A3*, c.925+20_926-48del; g.8061_8161del that has been previously identified in several patients with HHRH [19,31,32]. This deletion is pathogenic or likely pathogenic according to Varsome and it is found with total allele frequencies of 0.000294 and 0.000284 in exomes and genomes, respectively. No other pathogenic or potentially pathogenic variants were detected in the rest of the genes included in the panel. The c.1361C>T; p.(T454M) variant was confirmed after PCR amplification and Sanger sequencing of *SLC34A1* exon 12 (Figure 3A). Amplification and sequencing of this exon in the rest of the family members showed that both of the patient’s parents were heterozygous carriers of the missense variant and the patient’s sister presented the wild-type (WT) sequence (Figure 3A,D). On the other hand, family members were also tested for the presence of the 101 bp deletion; PCR amplification and agarose gel electrophoresis of the region between exons 9 and 10 of *SLC34A3* revealed that the patient inherited this mutation from his father who was also a heterozygous carrier (Figure 3B,C). The rest of the family members did not have the deletion.

### 2.3. In Silico Assessment of Variant c.1361C>T; p.(T454M)

**Figure 4:**
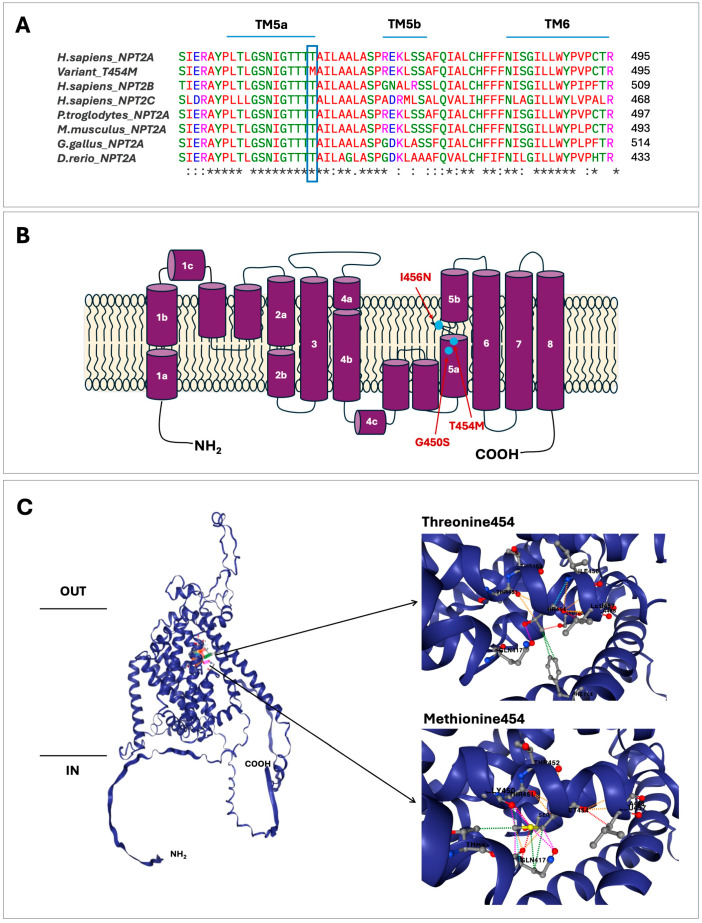
Location and effect of the threonine to methionine change in the NaPi-IIa protein. (**A**) Multiple alignments of NaPi-IIa protein sequences with a subset of vertebrate orthologues and other sodium/phosphate transporters. Residues that are conserved at 454 positions are marked with a rectangle. TMDs 5a, 5b and 6 are denoted by blue lines. An asterisk denotes a single, fully conserved residue. A colon denotes conservation between groups of strongly similar properties. A full stop indicates conservation between groups of weakly similar properties. No symbol indicates no conservation. (**B**) Secondary structure of NaPi-IIa protein (adapted from [2]). TMDs 1 to 8 are indicated. The locations of the novel variant, T454M, and the previously described variants, G450S and I456N [33,34], are marked with blue circles. (**C**) Predicted three-dimensional model of NaPi-IIa based on the AlphaFold DB model of human NaPi-IIa (AF-Q06495-F1-v4; UniProt ID Q06495), showing the location of T454M. The specific interactions between threonine and the mutant methionine at position 454 with neighboring amino acid residues were determined by DynaMut2.

According to the gnomAD database variant p.(T454M) is very rare, with a total allele frequency in exomes of 0.0000239 (it is not found in genomes). This variant appears in ClinVar as a variant of uncertain significance according to ACMG/AMP classification. The bioinformatic assessment could suggest a pathogenic effect (Table 2). The Franklin tool applied two criteria, PM2 and PP3, and VarSome applied the PP3 criteria. Furthermore, CADD scores provided additional evidence in support of the hypothesis of a pathogenic effect. Another commonly used prediction tools like PolyPhen-2, SIFT and MutPred2 gave a high score for this variant suggesting that it has a deleterious impact on the NaPi-IIa protein (Table 2). Evaluation of the NaPi-IIa protein with Clustal Omega demonstrated that amino acid residue threonine 454 is highly conserved in evolution suggesting its functional importance (Figure 4A). HOPE and Missense 3D tools showed that the change from threonine to methionine at position 454 results in significant differences in size, charge, and hydrophobicity. Methionine is a nonpolar amino acid, while threonine is polar and neutral at physiological pH, plus the methionine residue is larger and more hydrophobic than the threonine residue. These changes can lead to a protuberance and the loss of hydrogen bonds. The DynaMut2 prediction tool enabled us to observe the specific interactions between T454M and the nearest amino acids in the NaPi-IIa protein (Figure 4C). The presence of methionine eliminates interaction with F114, S168 and I456, while introducing numerous interactions that highlight hydrophobic and steric clashes (Figure 4C and Figure S1), which agrees with the HOPE predictions. On the other hand, DynaMut2 predicts a stability change for the mutant protein (ΔΔG was −0.27 kcal/mol), indicating a destabilizing effect. All these data suggest that the p.(T454M) variant could be a pathogenic mutation.

## 3. Discussion

Using exome sequencing analysis of a panel of genes associated with nephrolithiasis and nephrocalcinosis, we have identified a novel homozygous missense mutation, c.1361C>T; p.(T454M) located in the *SLC34A1* gene and a previously described heterozygous 101 bp deletion in the *SLC34A3* gene of a six-month-old girl with failure to thrive, polyuria, nephrocalcinosis, hypercalcemia, phosphate levels in the lower limit and elevated levels of 1,25-(OH)_2_D_3_. Results of the genetic analysis of the patient and their relatives revealed that the patient had inherited the *SLC34A1* mutation from both parents, whereas the *SLC34A3* deletion segregated from the paternal allele. *SLC34A1* encodes the sodium-phosphate cotransporter NaPi-IIa, which is a member of the solute carrier family 34, the main transporter involved in phosphate reabsorption in the renal tubule [2,17]. According to the Human Gene Mutation Database (HGMD) and recent publications, 40 missense mutations have been identified in *SLC34A1* gene [25,35,36,37,38,39]. The new *SLC34A1* missense variant replaces threonine, a neutral and polar amino acid, with methionine, a neutral and non-polar amino acid, at residue 454 on the NaPi-IIa protein. Our bioinformatics analyses indicate that this amino acid change could have a deleterious effect on protein structure/function. Pathogenic variants of *SLC34A1* cause IIH type 2, a rare tubulopathy associated with phosphate reabsorption defects [16,17]. The resulting hypophosphatemia produces a reduction in serum levels of FGF23 [40]. Consequently, these effects result in elevated 1,25-(OH)_2_D3 levels and hypercalcemia, as evidenced in our patient (Table 1). A renal ultrasound showed medullary nephrocalcinosis another characteristic of IHH patients (Figure 2C) [16].

The most recent model of the NaPi-IIa protein, based on the crystal structure of *Vibrio cholerae* sodium-dicarboxylate acid transporter, indicates that this protein includes eight transmembrane domains (TMDs), a large extracellular loop, and intracellular amino and carboxy ends [2,41] (Figure 4B). Many of *SLC34A1* mutations affect residues within the predicted highly conserved transmembrane domains (TMDs) of NaPi-IIa [17,24,39]. Functional studies with missense mutations in several heterologous expression systems have shown decreased phosphate uptake by the NaPi-IIa cotransporter when compared to the WT protein, which seems to be due to impaired trafficking of the mutant NaPi-IIa protein to the membrane [16,27,33,42,43].

The p.(T454M) mutation found in our patient affects a highly conserved residue of TMD5, which forms the Na3 binding site in the NaPi-IIa protein, probably impairing phosphate transport (Figure 4A,B) [40]. A previously described heterozygous mutation p.(I456N) that affects a nearby residue also in TMD5 has been shown to result in a trafficking defect [33]. This mutation was identified in a 26-year-old patient with hypophophatemia, renal phosphate wasting and nephrolithiasis. The expression of this mutant protein in *Xenopus oocytes* and a renal epithelium cell line showed significantly reduced phosphate transport compared to the normal NaPi-IIa protein. Fluorescent microscopy studies revealed that the p.(I456N) mutant protein fails to reach the membrane [33]. Additionally, a high-throughput exon sequencing analysis in an international cohort of 143 individuals with nephrolithiasis or isolated nephrocalcinosis detected another nearby heterozygous mutation, p.(G450S), also in TMD5 of NaPi-IIa, in a 7-year-old patient with nephrolithiasis [34]. All these findings indicate that the novel *SLC34A1* variant, p.(T454M), we identified alters the function of the NaPi-IIa protein. It is difficult to establish the clinical relevance of the p.(T454M) mutation in comparison to the other two mutations, p.(I456N) and p.(G450S), because our patient is homozygous for the p.(T454M) mutation, whereas the other two patients are heterozygous for their respective mutations. *SLC34A1* mutations tend to produce a more severe and earlier-onset phenotype when both alleles are affected [17,22]. Accordingly, our patient presented symptoms of the disease at only 6 months of age. As mentioned before, the pathogenic 101 bp deletion detected in the *SLC34A3* gene, which encodes the sodium-dependent phosphate cotransporter NaPi-IIc, of our patient and her father has been previously detected in several patients with HHRH in homozygous, heterozygous and compound heterozygous states [19,31,32]. Molecular analysis by RT-PCR and DNA sequencing of the products have shown that the 101 bp deletion alters the splicing of the *SLC34A3* pre-mRNA since it affects the minimal intron size needed for proper pre-mRNA splicing [31]. The region between exon 9 and intron 9 contains two homologous sequence repeats (direct repeats), which are probably involved in the generation of the deletion (Figure 3B,C) [31].

The patient’s father carried both the *SLC34A1* and *SLC34A3* mutations in heterozygosis, he had hypophosphatemia but was asymptomatic. Heterozygous carriers of *SLC34A3* variants show a milder clinical manifestation in comparison to those who are homozygous or compound heterozygous variants [22,44,45,46]. Several studies have shown that many heterozygous carriers have hypercalciuria, reduced renal phosphate reabsorption and a higher risk for renal calcifications than the general population [26,47,48]. We hypothesize that the heterozygous 101 bp deletion detected in the *SLC34A3* gene of our patient may increase the risk of renal calcifications and the severity of other symptoms caused by homozygous *SLC34A1* mutation. However, other contributory factors such as environmental, dietary, or polygenic, could also influence the phenotype of IHH patients [23,24]. IHH is generally considered to be an autosomal recessive disorder. Consequently, future descendants in the family will have a 25% probability of inheriting the mutated gene from both parents and developing the disease, and a 50% probability of inheriting one mutant gene and being carriers. Genetic counseling and carrier testing are recommended for family members in order to facilitate informed discussion regarding future reproductive decisions.

As far as we know, this is the first report of the combination of a *SLC34A1* homozygous mutation and a heterozygous *SLC34A3* mutation in the same patient. Recently, Gordon et al. [36] described another unusual case of an 11-year-old girl with autosomal dominant HHRH-carrying heterozygous mutations in *SLC34A1* and *SLC34A3* genes with rickets, short stature, hypercalciuria, and bilateral nephrolithiasis. They suggested a cooperative role of NaPi-IIa and NaPi-IIc transporters in regulating systemic phosphate homeostasis and implicating a novel digenic mechanism for HHRH. Three other cases with one heterozygous variant in the *SLC34A3* gene and a second variant in the *SLC34A1* or *CYP24A1* genes have been described, also suggesting the interaction between their protein products [25,49].

### Conclusions

In summary, the novel homozygous missense mutation, p.(T454M), identified in the *SLC34A1* gene of our patient, together with the clinical manifestations and biochemical data, confirmed the diagnosis of IIH type 2. While the pathogenic effect of this variant is suggested by clinical evidence, functional studies are required to confirm it, which is a limitation of the present study. Mutation p.(T454M) affects transmembrane domain 5 of the NaPi-IIa protein, which is involved in substrate binding, resulting in impaired phosphate transport. This is the first report of the digenic inheritance of a homozygous missense mutation in *SLC34A1* and a heterozygous mutation in *SLC34A3*. Our results highlight the importance of performing exome analysis during the differential diagnosis of hypercalcemia in children, and we recommend its use in the diagnosis of these rare phosphate-wasting disorders.

## 4. Materials and Methods

This study was carried out following protocol CHUNSC_2023_113 approved by the Ethics Committee of the *Complejo Hospitalario Universitario de Canarias* (Santa Cruz de Tenerife, Spain) and written informed consent for the genetic analysis was obtained from the patient’s parents in accordance with the Declaration of Helsinki. Blood and urine samples were collected for biochemical and genetic analysis.

### 4.1. Biochemical Determinations

Serum and urine biochemistry was performed in the clinical analysis laboratory of the Hospital General of Fuerteventura using conventional biochemical methods on Abbott Alinity analyzers (Abbott Core Laboratory Systems, Lake Forest, IL, USA). For serum and urine creatinine determinations, the CREA2 assay was used, which applies the Jaffé method [50]. PTH levels were measured using the intact PTH assay, a chemiluminescent microparticle immunoassay (CMIA). Serum 25-(OH) vitamin D was determined using a chemiluminescent microparticle assay. Both were performed on the Alinity i module. Serum 1,25-(OH)_2_D_3_ was measured by radioimmunoassay in an external laboratory (Reference Laboratory S.A, Barcelona, Spain).

### 4.2. Genetic Analysis

Exome and Sanger sequencing were used for the mutation analysis. Thirty genes associated with nephrolithiasis and nephrocalcinosis (Illumina Exome 2.5 Panel) were analyzed by Next Generation Sequencing (Reference Laboratory Genetics, Barcelona, Spain). The following genes were included in the panel: *ADCY10*, *AGXT*, *APRT*, *ATP6V0A4*, *ATP6V1B1*, *CA2*, *CASR*, *CLCN5*, *CLDN16*, *CLDN19*, *CYP24A1*, *FAM20A*, *GRHPR*, *HNF4A*, *HOGA1*, *HPRT1*, *KCNJ1*, *NHERF1*, *OCRL*, *SLC12A1*, *SLC22A12*, *SLC26A1*, *SLC2A9*, *SLC34A1*, *SLC34A3*, *SLC3A1*, *SLC4A1*, *SLC7A9*, *VDR*, and *XDH.* Variants found were confirmed by Automatic DNA sequencing. Genomic DNA from family members and control was extracted from peripheral blood samples using the NuceloSpin Blood kit according to the manufacturer’s instructions (Macherey-Nagel, Düren, Germany). Exon 12 of *SLC34A1* was PCR amplified using primers and conditions previously described [27]. To analyze the region between exons 9 and 10 of *SLC34A3* we used the following PCR primer pairs: 5′ TGCCCACTGAGCCTGTCC 3′ (exon 9 P1 forward) and 5′ CAGGTCCGTGAGCTCCGT 3′ (exon 10 P1 reverse) or 5′ CTGAGCCTGTCCTGAGTCC 3′ (exon 9 P2 forward) and 5′ CTCCGTGCCCGCAAACAG 3′ (exon 10 P2 reverse). These primers were designed using Primer3 (https://primer3.ut.ee/) (accessed on: 27 November 2024) and SnapGene software (www.snapgene.com) (accessed on: 27 November 2024). The amplification conditions were 35 cycles at annealing temperature of 62 °C, using the KAPA2G Robust HotStart (Kapa Biosystems—Hoffman-La Roche, Wilmington, MA, USA). PCR products were analyzed by agarose gel electrophoresis, and the fragments were purified using the NucleoSpin Gel and PCR Clean-up kit (Macherey-Nagel, Düren, Germany). DNA sequencing was performed by Macrogen Spain (Madrid, Spain).

Variants were localized by comparison to the respective reference sequences, GenBank accession number NM_003052.5; (transcript ENST00000324417.6) and NM_001177316.2 (transcript ENST00000673835.1) for *SLC34A1* and *SLC34A3*, respectively, with the Basic Local Alignment Search Tool (BLAST) (https://blast.ncbi.nlm.nih.gov/Blast.cgi) (accessed on: 10 October 2024) [51]. The presence of the variants identified was queried in several databases of genetic variants, including the Genome Aggregation Database v4.1 (gnomAD v4.1) (https://gnomad.broadinstitute.org/) (accessed on: 10 October 2024) [52], MutationTaster2021 (https://www.genecascade.org/MutationTaster2021/ (accessed on: 10 October 2024) [53], ClinVar (https://www.ncbi.nlm.nih.gov/clinvar/) (accessed on: 4 April 2025) [54] and the Human Gene Mutation Database (http://www.hgmd.cf.ac.uk/ac/index.php) (accessed on: 10 October 2024) [39].

### 4.3. In Silico Prediction Analysis

To assess the pathogenicity of the novel missense variant identified in the *SLC34A1* gene, the following bioinformatics tools were used: VarSome (https://varsome.com/) (accessed on: 14 October 2024) [55], Combined Annotation Dependent Depletion (CADD) https://cadd.gs.washington.edu/) (accessed on: 17 July 2025) [56] and Franklin (https://franklin.genoox.com/clinical-db/home) (accessed on: 14 October 2024). These platforms classify variants as pathogenic, likely pathogenic, benign, likely benign, or of uncertain significance according to the American College of Medical Genetics and Genomics (ACMG) and Association for Molecular Pathology (AMP) guidelines [57]. Furthermore, another three bioinformatics tools were used to predict the impact of the amino acid substitution on the NaPi-IIa protein: PolyPhen-2 (PolyPhen-2: http://genetics.bwh.harvard.edu/pph2/bgi.shtml) [58], SIFT (https://sift.bii.a-star.edu.sg/) [59] and MutPred2 (http://mutpred.mutdb.org/) [60], all accessed on: 24 October 2024). Variants are classified as benign or potentially damaging according to each algorithm criteria. Clustal Omega (https://www.ebi.ac.uk/Tools/msa/clustalo/) (accessed on: 25 October 2024) [61] was used to assess the conservation of the residue affected by the variant by aligning the sequence of the human NaPi-IIa protein with several vertebrate orthologues (*Pan troglodytes, Mus musculus*, *Gallus gallus*, *and Danio rerio*), as well as with other human sodium/phosphate co-transporters (NaPi-IIb and NaPi-IIc).

### 4.4. Computational Protein Modeling

AlphaFold (model ID: AF-Q06495-F1-v4; UniProt ID: Q06495) (https://alphafold.ebi.ac.uk/) (accessed on: 15 October 2024) [62], an artificial intelligence-based tool that predicts protein structures directly from amino acid sequences, was used to predict the three-dimensional structure of the human NaPi-IIa protein. To evaluate the potential impact of the amino acid substitution on the protein structure, we used HOPE (https://www3.cmbi.umcn.nl/hope/) (accessed on: 21 October 2024) [63] and Missense3D (https://missense3d.bc.ic.ac.uk/) (accessed on: 28 October 2024) [64]. We also used DynaMut2 (https://biosig.lab.uq.edu.au/dynamut2/) (accessed on: 19 November 2024) [65] to evaluate the variant’s impact on the structure and thermodynamic stability of the protein. This tool provides a consensus prediction of the change in Gibbs free energy (ΔΔG); negative values indicate a destabilizing effect. It also allows interactions formed by WT and variant residues to be visualized and compared. For the Missense3D and DynaMut2 analyses, we used the AlphaFold-predicted model as there is currently no experimentally determined NaPi-IIa structure available in the Protein Data Bank.

## Figures and Tables

**Table 1:** Evolution of biochemical parameters measured in the patient’s blood and urine.

	Age (Months)	7	10	16	22	28	Reference Values
Blood	Creatinine (mg/dL) *	0.47 (+0.91)	0.42 (0.00)	0.42 (–1.25)	0.44 (–0.75)	0.41 (–1.50)	15 days-<1 year: 0.31–0.53. 1–2 years: 0.39–0.55.
	Calcium (mg/dL)	11.2 (+3.5)	11.5 (+4.25)	10.7 (+2.25)	10.4 (+1.50)	10.0 (+0.50)	10 days-2 years: 9–10.6
	Phosphate (mg/dL)	4.6 (–2.31)	4.5 (–2.46)	4.2 (–2.60)	3.9 (–3.20)	3.2 (–4.60)	0–1 year: 4.8–7.4 1–5 years: 4.5–6.5
	PTH ^a^ (pg/mL)	13.8 (–2.42)	16 (–2.30)	-	12.2 (–2.50)	-	22.0–101.0
	25-(OH) vitamin D (ng/mL)	58.6 (+0.54)	54.0 (+0.13)	36.1 (–1.46)	-	31.1 (–1.90)	30–75
	1,25-(OH)_2_D_3_ (pg/mL)	78 (+4.82)	43 (+0.71)	74 (+4.35)	-	71 (+4.00)	20–54
Urine	TRP ^b^ (%) **	75.29 (–0.41)	69.68 (–1.07)	54.69 (–2.86)	61.32 (–2.07)	64.22 (–6.61)	0–2 years: 78.7 ± 8.4 >2 years: 92 ± 4.2
	Calcium/Creatinine (mg/mg)	0.33 (+0.10)	0.47 (+1.08)	0.51 (+2.45)	0.15 (–0.88)	0.31 (+4.44)	0.1–0.5 years: 0.03–0.80 0.6–1 year: 0.03–0.60 1–2 years: 0.03–0.46 2–3 years: 0.02–0.20
	Protein/Creatinine (mg/mg)	0.56	0.44	-	0.00	0.41	<2 years: <0.5 >2 years<0.2
	V/GFR ^c^ (%)	2.70	2.57	2.94 (+10.68)	3.21 (+11.91)	2.05 (+6.64)	>1 year: 0.59 ± 0.22

Values are expressed as absolute measurements with corresponding z-scores relative to these references. ^a^ PTH: parathyroid hormone; ^b^ TRP: tubular reabsorption of phosphate; ^c^ V/GFR: urine output per 100 mL of glomerular filtrate; -: not measured. Reference values were obtained from [28]. * Creatinine values were based on Caliper values for Abbott standardized Jaffé creatinine [29]. ** TRP values [30].

**Table 2:** Bioinformatics predictions of pathogenicity for the *SLC34A1* missense variant.

VarSome ^a^	Franklin ^b^	CADD ^c^	Polyphen2 ^d^	SIFT ^e^	MutPred ^f^
PP3	PM2, PP3	32	1.00	0.00	0.818

^a^ PP3 (VarSome): pathogenic moderate. ^b^ PM2: Extremely low frequency in gnomAD population databases; PP3: pathogenic moderate; score: 0.878. Computational prediction tools unanimously support a deleterious effect on the gene. ^c^ CADD phred score: ranges from 1 to 99; higher values indicate more deleterious. The highest score cutoff recommended by the authors is 20. ^d^ Polyphen-2 score: Scores range from 0 to 1 (0, benign; 1, probably damaging). ^e^ SIFT score (SIFTori) ranges from 0 to 1. The smaller the score the more likely the variant has damaging effect (0, pathogenic supporting). ^f^ MutPred scores range from 0 to 1. The larger the score the more likely the variant has damaging effect. (0.818, pathogenic moderate).

## Data Availability

The data presented in this study are available on request from the corresponding author.

## Supplementary Materials

The following supporting information can be downloaded at: https://www.mdpi.com/article/10.3390/ijms26178541/s1.
