# Supplementary material for: Identification of a Novel Homozygous SLC34A1 Missense Mutation and a Heterozygous SLC34A3 Deletion in an Infant with Nephrocalcinosis, Failure to Thrive, and Hypercalcemia
Source: Int J Mol Sci. 2025 Sep 2;26(17):8541. doi: 10.3390/ijms26178541 (PMC12428849; doi:10.3390/ijms26178541)

Supplementary Figure S1. Schematic representation of the molecular interactions formed by WT threonine (top) and mutant methionine (bottom) at position 454 **of the** NaPi-IIa **protein** with surrounding amino acid residues. Dashed lines indicate interaction types: hydrophobic (red), polar (orange), van der Waals (green), and clash (blue/purple). Threonine 454 engages in polar interactions via its hydroxyl group, whereas methionine 454 forms additional hydrophobic and van der Waals contacts, as well as steric clashes, potentially affecting local protein conformation. Atom labels indicate the interacting atoms: **O** (oxygen), **N** (nitrogen), and **C** (carbon).

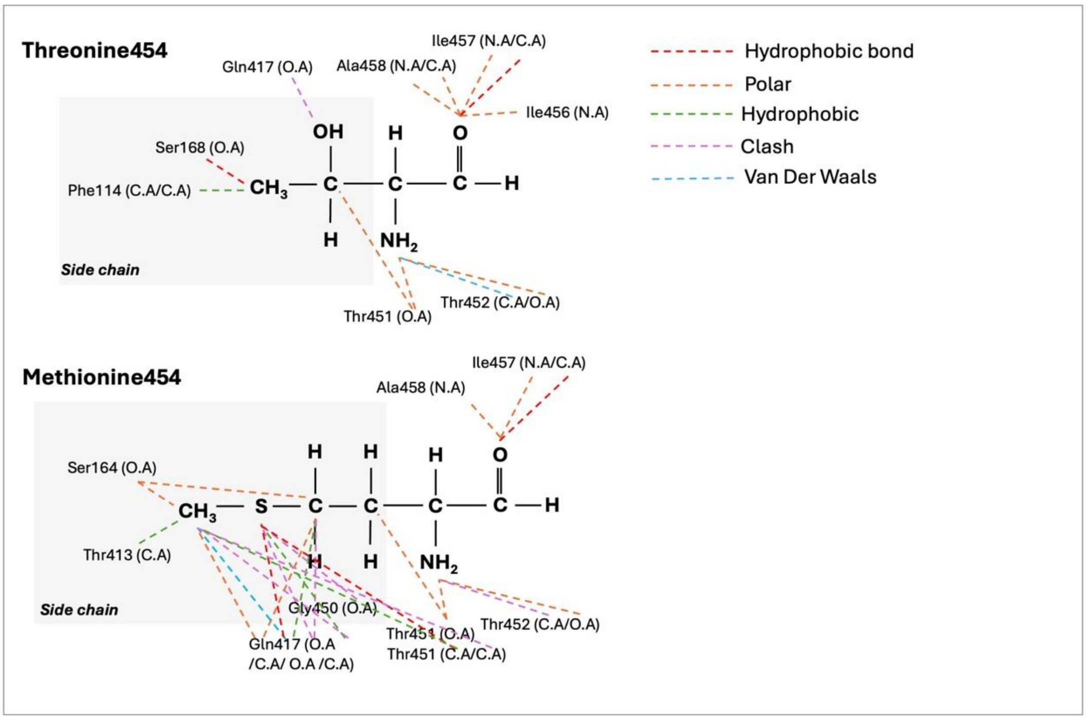

Supplement: Supplementary file 1 [file ijms-26-08541-s001.zip › ijms-3824448-supplementary.pdf]
